# Expression of CISH, an Inhibitor of NK Cell Function, Increases in Association with Ovarian Cancer Development and Progression

**DOI:** 10.3390/biomedicines11020299

**Published:** 2023-01-21

**Authors:** Jasmin C. Acosta, Janice M. Bahr, Sanjib Basu, James T. O’Donnell, Animesh Barua

**Affiliations:** 1 Department of Anatomy and Cell Biology, Rush University Medical Center, Chicago, IL 60612, USA; 2 Department of Animal Science, University of Illinois at Urbana-Champaign, Urbana, IL 61801, USA; 3 Department of Internal Medicine, Rush University Medical Center, Chicago, IL 60612, USA; 4 Department of Obstetrics and Gynecology, Rush University Medical Center, Chicago, IL 60612, USA; 5 Department of Pathology, Rush University Medical Center, Chicago, IL 60612, USA

**Keywords:** ovarian cancer, immune inhibition, CISH, cellular stress, cancer progression

## Abstract

Epithelial ovarian cancer (OVCA), a fatal malignancy of women, disseminates locally. Although NK cells mount immune responses against OVCA, tumors inhibit NK cells, and the mechanism is not well understood. Cytokines stimulate NK cells; however, chronic stimulation exhausts them and induces expression of cytokine-inducible SH2-containing protein (CISH). Tumors produce anti-inflammatory cytokine interleukin (IL)-10 which may induce NK cell exhaustion. The goal of this study was to examine if CISH expression in NK cells increases during OVCA development and to determine the mechanism(s) of OVCA-induced CISH expression in NK cells. Normal ovaries (*n* = 7) were used for CISH, IL-10 and GRP78 expression. In tumor ovaries, CISH was examined in early and late stages (*n* = 14 each, all subtypes) while IL-10 and GRP78 expression were examined in early and late stage HGSC (*n* = 5 each). Compared to normal, the population of CISH-expressing NK cells increased and the intensity of IL-10 and GRP78 expression was significantly higher in OVCA (*p* < 0.05). CISH expression was positively correlated with IL-10 expression (r = 0.52, r = 0.65, *p* < 0.05 at early and late stages, respectively) while IL-10 expression was positively correlated with GRP78 expression (r = 0.43, r = 0.52, *p* < 0.05, respectively). These results suggest that OVCA development and progression are associated with increased CISH expression by NK cells which is correlated with tumor-induced persistent cellular stress.

## 1. Introduction

The aggressive rates of growth and non-specificity of symptoms during early stages and the lack of an early detection test are the reasons why the majority of ovarian cancer (OVCA) cases are diagnosed at late stages [1]. The five-year survival rates of patients decrease to less than 50% when OVCA is detected at late stages as opposed to more than 90% if detected at earlier stages [2]. Cytoreductive surgery followed by chemo- and radio- therapies are currently used treatment modalities. Tumors, in most cases, develop resistance to currently available chemotherapies leading to frequent recurrence of OVCA, making it a lethal malignancy of women [3]. In addition, chemotherapeutics are toxic to normal uninvolved tissues while radiotherapies are associated with unwanted injuries; thus, immunotherapies appear to be a safer option for OVCA patients [4].

OVCA disseminates in the peritoneal cavity through diffusion as well as through the hematological route [5]. Thus, local immunity plays a crucial role in preventing OVCA development and progression. Although the immune system mounts an immune response against a developing tumor, the tumor frequently evades the anti-tumor immune response and continues to grow and metastasize. Previous studies suggested that tumors evade an immune response by either immunosuppression or escaping recognition by immune cells [6,7]. Immunotherapies including monoclonal antibodies targeting CTLA-4 (cytotoxic T-lymphocyte associated protein 4), inhibitors of the immune checkpoint programmed cell death protein-1 (PD-1) which is a receptor expressed on the surface of T and B-lymphocytes, and its ligand PD-L1, have been developed [8]. Unfortunately, the effectiveness and response of these immunotherapies was limited to only 10–20% or 20–50% against CTLA-4 or PD-1/PD-L1, respectively [9]. While this effectiveness was limited in cancers such as head and neck cancer and B cell lymphoma, it was even lower against OVCA, highlighting the urgent need for the development of additional immunotherapies [9]. As OVCA disseminates through diffusion in the peritoneal cavity, local immune functions play an important role in preventing tumor growth and metastasis.

NK cells are members of the innate immune system and provide the first line of defense against an invading pathogen and can kill tumor cells directly without the need of an antigen presenting cell, including MHC-I and MHC-II molecules [10]. NK cells recognize tumor cells through their NKG2D receptor binding to its ligand, MHC class I-polypeptide related sequence A or B (MICA/MICB) which are expressed on the surface of tumor cells [11]. However, tumor cells are able to evade recognition by NKG2D-expressing NK cells by shedding the MICA/B ligand from their surface and allowing them to continue to grow and metastasize [12]. The ovarian tumor-induced suppression of NK cell immunity has been reported both in OVCA patients and hens, a preclinical model of spontaneous OVCA [13,14,15,16]. Emerging information suggests that tumors may use additional mechanism(s) for the suppression of anti-tumor functions of NK cells. The exhaustion of NK cells may be one of such additional mechanisms.

Cytokines including interleukin 15 (IL-15) are used to stimulate NK cells, and emerging information shows that prolonged stimulation may exhaust NK cells by inducing cytokine inducible SH2-containing protein (CISH) [17]. The induction of CISH, a member of the suppressor of cytokine signaling (SOCS) family, may suppress cytokine-induced stimulation of NK cells [18]. Ovarian tumors have been reported to secrete cytokines including IL-10 and IL-15 [19,20]. However, the expression of CISH as a mechanism of suppression of NK cell immunity against ovarian tumors is not known. IL-10 is an anti-inflammatory cytokine and is produced by activated immune cells [21]. Its anti-inflammatory properties play a role in limiting host immune responses to pathogens and prevent the damage of host tissues by maintaining normal tissue homeostasis [22]. IL-10 is considered to be a potent negative regulator of proliferative and inflammatory responses of various immune cells including T cells, B cells, monocytes and macrophages [23]. It is possible that IL-10 may also be associated with reducing the anti-tumor function of NK cells by inducing CISH expression. In addition, the factor(s) responsible for tumor-induced secretion of IL-10 is not known. Tumor development and progression is accompanied with chronic cellular stress, which may be a reason for IL-10 secretion by malignant cells. To survive from chronic stress, malignant cells secrete glucose-regulated protein 78 (GRP78, an endoplasmic reticulum resident chaperone), a marker of cellular stress [24,25,26]. It is possible that GRP78 may be associated with increased secretion of IL-10 by ovarian tumors and may expose NK cells homing to the tumor (intra-tumoral) as well as in the tumor vicinity. The goal of this study was to examine whether the population of CISH-expressing NK cells increased in ovarian tumors during OVCA development and progression and whether CISH-expression was associated with GRP78 and IL-10 levels.

## 2. Materials and Methods

### 2.1. Clinical Specimens

Archived clinical specimens were collected from the Department of Pathology at Rush University Medical Center (RUMC); normal ovarian tissues and fimbria of the fallopian tube were archived from patients who underwent surgery due to non-ovarian causes and tumor tissues were archived from patients with OVCA following surgery. All specimens were obtained under and used as per the protocols approved by the Institutional Review Board (IRB) at Rush University Medical Center, Chicago, IL, USA. Paraffin-embedded tissue sections were made (5 µm thickness) and stained with hematoxylin & eosin for histopathology and for immunohistochemistry (IHC) as previously reported [27]. All clinical information, including tumor stages, was obtained from the final pathology report from the Department of Pathology at RUMC, Chicago, IL. The staging of tumors were performed during surgery and were staged in the early stage (stage IA, IB, IC and stage IIA, IIB) as well as the late stage (Stage III and IV inclusive of all subgroups) according to the FIGO (International Federation of Gynecology and Obstetrics) classifications for ovarian cancer [28]. All specimens were obtained from patients during primary cytoreductive surgery before patients received any therapy as reported earlier [29].

### 2.2. Immunohistochemistry

Representative specimens, including normal ovaries (*n* = 7) and ovarian malignant tumors from patients with OVCA at early and late stages (*n* = 5 for each), were selected from the archive mentioned above and paraffin sections were used for immunohistochemistry as previously reported [16]. In brief, sections were deparaffinized using a graded series of ethanol and were rinsed in deionized (DI) water. Antigens in the sections were retrieved by heating the sections in citrate solution (pH 6.0) as per the manufacturer’s recommendation reported earlier [30]. Endogenous peroxidases in sections were blocked by incubating in ice-cold 0.3% H_2_O_2_ in a methanol solution. Sections were then incubated in a normal horse serum (Vector Laboratories, Burlingame, CA, USA) to block non-specific binding and were then incubated overnight with primary antibodies, including anti-CISH (*n* = 5 ovarian HGSC, *n* = 3 per group of endometrioid, mucinous and clear cell subtypes per stage) (Abcam, Cambridge, MA, USA), or anti-IL-10 (*n* = 5 per stage) (Millipore Sigma, St. Louis, MO, USA), or anti-GRP78 (*n* = 5 per stage) (Abcam, Cambridge, MA, USA) at a 1:100 dilution. After overnight incubation, the sections were washed in phosphate buffered saline (PBS, 3 × 5 min) and incubated with biotinylated secondary antibody for 1 h. After washing in PBS (3 × 5 min), the sections were incubated with peroxidases conjugated with avidin for 1 h and washed with PBS (3 × 5 min). Immunoreactions on sections were then visualized with 3,3′-Diaminobenzidine (DAB) containing H_2_O_2_ under a light microscope. The sections were then washed in DI water and counterstained with hematoxylin and dehydrated with a graded series of ethanol and xylene. The sections were then mounted with mounting media, covered with a cover slip and oven dried. They were then examined under a light-microscope attached to a computer-assisted software program (MicroSuite™ version 5, Olympus American Inc., Center Valley, PA, USA). Images from at least 5 areas of a section with highest staining were taken at 40× magnification and archived for later analysis. For all markers, the primary antibody was omitted in control staining and immunoreactions were not observed on the control sections. Details of antibody information are provided in Supplementary Table S1 and details for sample sizes are provided in Supplementary Table S2. Immunohistochemical staining in IL-15, pan-Cytokeratin and CD10 expression were performed similarly as mentioned above. In addition, the expressions of CISH and GRP78 by normal fimbria of the fallopian tube were also examined (Supplementary Figure S1).

#### Counting of Immunopositive Cells or Intensity of Immunostaining

The population of immunopositive cells was counted as reported earlier [29]. Briefly, for CISH, 3-5 areas with high populations of immunoreactive cells in a section at 40× magnification were counted. Immunopositive cells in the intra-tumoral and tumor stroma areas were counted. Pan-cytokeratin staining and CD10 marker staining were used as epithelial cell and stromal cell markers, respectively (Supplementary Figure S2). The mean population of CISH-expressing cells in 20,000 µm^2^ area of tissue in each section was determined. Similarly, intensity of IL-10 and GRP78 expression in 20,000 µm^2^ area of tissue was determined as arbitrary values as reported earlier [31].

### 2.3. Immunoblotting

The expression of CISH, IL-10 and GRP78 protein was examined by one dimensional Western blotting as reported earlier [32]. Briefly, tissue homogenate was collected from representative specimens including normal ovaries, early stage and late stage OVCA. Proteins were separated by gel electrophoresis and transferred to a nitrocellulose membrane. The membranes were incubated with the primary antibodies mentioned above at 1:500 (CISH) or 1:1000 (for IL-10 or GRP78) dilutions. Corresponding secondary antibodies conjugated with horse-radish peroxidase were used (Thermo Fisher Scientific, Waltham, MA, USA). Immunoreactions on the CISH and GRP78 membranes were detected as chemiluminescence product (ECL Immobilon Forte Western HRP Substrate, Millipore Sigma, Burlington, MA, USA) and images were captured using a ChemiDoc XRS (BioRad Laboratories, Hercules, CA, USA) system. For IL-10, after incubation with a primary antibody, the membrane was washed in phosphate-buffered saline (PBS) 3 times at 5-min intervals. After washing, the membrane was incubated in a secondary antibody (Horse Anti-mouse/Rabbit IgG (H + L), Biotinylated, R.T.U, Vector, Burlingame, CA, USA) for 1 h and then washed in PBS as mentioned earlier. The membrane was incubated in an enzyme (Streptavidin Peroxidase, R.T.U, Vector, Burlingame, CA, USA) for 1 h and then washed. The antigen-antibody reaction on the membrane was then visualized with 3,3-diaminobenzidine peroxidase substrate (DAB, Vector DAB Substrate Kit, Vector, Burlingame, CA, USA) and stopped with DI water [33]. Images were captured with a dual-lens camera system (Apple Inc., Cupertino, CA, USA) immediately after immunoreaction was stopped. Images were archived for later use. No immunoreactivity was observed in controls where proteins were omitted, anti-β-Actin was used as the loading control. Signal intensity of each immunoreactive band was determined using the analysis getIT!™ software (Olympus Soft Imaging Solutions Corporation, Lakewood, CO, USA) as previously reported [34]. Signal intensities are presented as an intensity ratio (mean ± SEM) (Supplementary Figure S3).

### 2.4. Reverse-Transcriptase Polymerase Chain Reaction (RT-PCR) and Quantitative-RT-PCR (q-RT-PCR)

All mRNA extraction and cDNA synthesis for gene expression studies were prepared from representative samples mentioned above as previously reported [34].

The expression of IL-10 and GRP78 in normal ovaries, early stage and late stage ovarian HGSC were examined by RT-PCR using β-actin as a control as reported previously [35]. qRT-PCR assays were performed using the Applied Biosystems Viia7 Real-Time PCR system and were analyzed by the associated computer software (Thermo Fisher Scientific, Waltham, MA, USA) as reported earlier [34]. The following primers were used in RT-PCR and qRT-PCR:

Primer sequences (5′ → 3′):
CISH-F: CTGCTGTGCATAGCCAAGACR: TAAGAACGTGCCTTCTGGCATIL-10-F: CCTGCCTAACATGCTTCGAGAR: TGGCAACCCAGGTAACCCTTGRP78-F: GCCTGTATTTCTAGACCTGCCR: TTCATCTTGCCAGCCAGTTGβ -Actin-F: CCACCATGTACCCTGGCATTR: GTACTTGCGCTCAGGAGGAG

### 2.5. Statistical Analysis

The differences in the population of CISH-expressing cells among normal ovaries, ovaries with tumor at early and late stages, as well as differences in intensities of IL-10 or GRP78 expression during OVCA development and progression, were analyzed using Graph-Pad Prism version 9.4.1 (GraphPad Software Inc., La Jolla, CA, USA) and assessed by one-way ANOVA or unpaired *t*-tests. Correlation analysis between CISH and IL-10 and between GRP78 and IL-10 was performed in GraphPad Prism version 9.4.1 (GraphPad Software Inc., La Jolla, CA, USA) where the Pearson correlation coefficient was computed at a 95% confidence interval. Significant differences in the population of immunopositive cells, intensities of immunohistochemical staining and signal intensities in immunoblotting or fold changes in gene expression among different groups are shown using letters (a, b, c, d), and different letters denote significant differences among groups. *p* values < 0.05 were considered significant. 

## 3. Results

### 3.1. Microscopic Features of Ovarian Malignant Tumors

**Figure 1:**
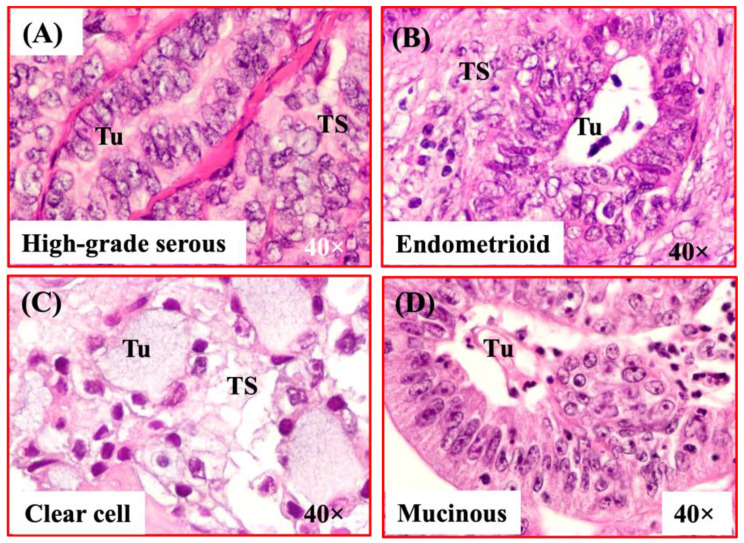
Microscopic presentation of epithelial ovarian cancer (OVCA). (**A**) Section of an ovarian high-grade serous carcinoma (HGSC). (**B**) Section of an endometrioid carcinoma. (**C**) Section of an ovarian clear cell carcinoma. (**D**) Section of an ovarian mucinous carcinoma. TS = Tumor stroma, Tu = Tumor. 40× = magnification.

Ovarian high-grade serous carcinoma appeared like a compact sheath of malignant cells containing large pleomorphic nuclei separated by fibromuscular tissue (Figure 1A). In some cases, it appeared as lace-like papillary structures while in other cases it appeared as labyrinth of slit-like glands. Ovarian endometrioid carcinoma showed confluent back-to-back glandular structures containing a single layer of epithelial cells with large nuclei and a sharp luminal lining (Figure 1B). Ovarian clear cell carcinoma showed vacuolated malignant cells containing larger nuclei and scanty cytoplasmic materials (Figure 1C). Ovarian mucinous carcinoma consisted of glandular structures containing a single layer of columnar-like epithelial cells and mucin-like luminal secretion. Nuclei in malignant cells in ovarian mucinous carcinoma are located distal from the basement membranes close to the apical surface of the cells (Figure 1D).

### 3.2. Changes in Population of Ovarian CISH-Expressing Cells during OVCA Development and Progression

**Figure 2:**
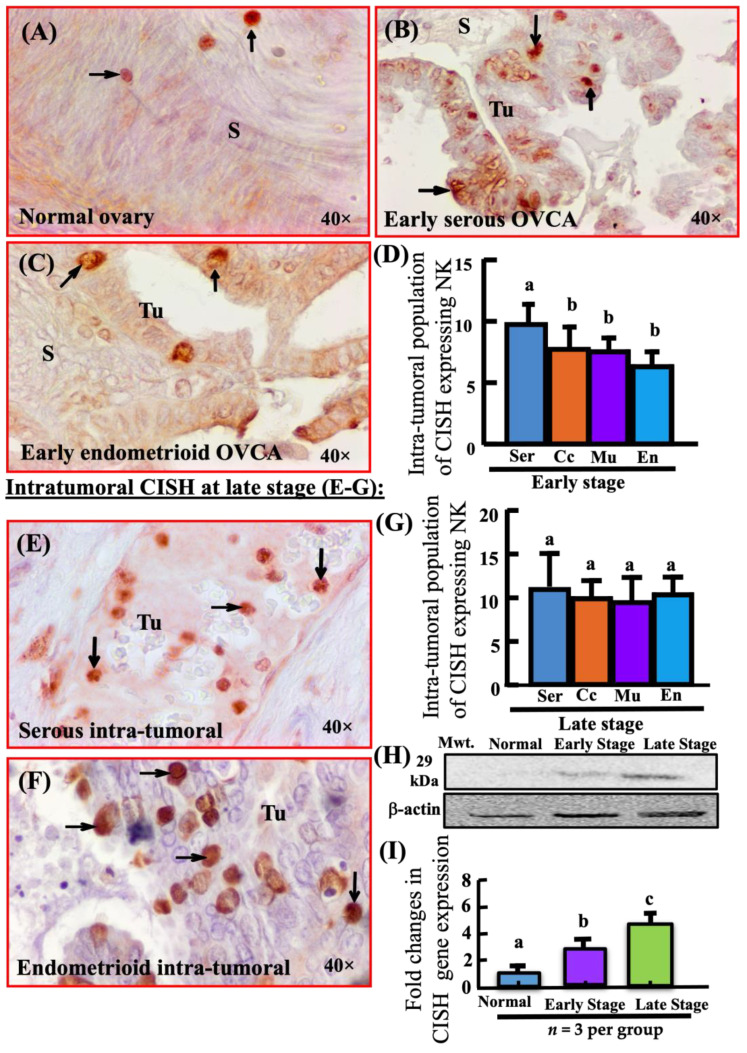
Localization of CISH-expressing cells in normal ovaries or ovaries with tumor. Intra-tumoral CISH-expressing cells in early stage OVCA (**A**–**D**). (**A**) Section of a normal ovary showing few CISH-expressing cells in ovarian stroma. (**B**) Section of a high-grade serous carcinoma (HGSC) showing CISH-expressing cells in the tumor. (**C**) Section of an endometrioid carcinoma at early stage showing few CISH-expressing cells in the tumor. (**D**) Changes in the population of CISH-expressing cells in different tumor types during OVCA development (early stage). Among different types, the population of CISH-expressing cells were significantly greater in HGSC (*p* < 0.05, *n* = 5) than other histological types; En, Mu and Cc (*n* = 3 for each group). Intra-tumoral CISH-expressing cells in late stage OVCA (**E**–**G**). (**E**) Section of a HGSC at late stage showing many intra-tumoral CISH-expressing cells. (**F**) Section of endometrioid OVCA at late stage showing many CISH-expressing cells. (**G**) Population of CISH-expressing cells in different tumor types; En, Mu, Cc (*n* = 3 for each group) during OVCA progression at late stages. Significant differences in the population of CISH-expressing cells were not observed among different types of epithelial OVCA at late stages. (**H**) Immunoblotting detected a protein band of approximately 29 kDa. The signal intensity of CISH protein expression was very weak or almost undetectable for normal ovaries, moderate for early stage HGSC and stronger for late stage HGSC. Additional information: Compared to normal, signal intensities of CISH expression were significantly high in early stage HGSC (normal vs. early, *p* < 0.01) and in late stage HGSC (normal vs. late stage, *p* < 0.01) (Supplementary Figure S3A). (**I**) As observed in CISH protein expression, expression of CISH mRNA increased significantly during OVCA development and progression (normal vs. early HGSC, *p* < 0.05; normal vs. late HGSC, *p* < 0.01, *n* = 3 per group). Bars with different letters (in (**D**,**G**)) are significantly different. En, Se, Mu and Cc = Endometrioid, Serous, Mucinous and Clear cell OVCA, respectively; S = Stroma; Tu = Tumor. Values for CISH-expressing cells are presented (in (**D**,**G**)) as mean ± SEM in 20,000 µm^2^ area of the tissue and bars with different letters are significantly different for population of CISH-expressing cells. 40× = magnification. Arrows indicate examples of immunopositive CISH-expressing cells.

In normal ovaries, few CISH-expressing cells were localized in the stroma (Figure 2A). In contrast, many CISH-expressing cells were located both inside the tumor (intratumor) as well as in the tumor stroma in early and late stages (Figure 2B,C,E,F). Among all four subtypes of epithelial OVCA at early stage, the population of intra-tumoral CISH-expressing cells was highest in ovarian high-grade serous carcinoma (HGSC) (mean ± SEM in 20,000 µm^2^ area, *n* = 5, *p* < 0.0001) as compared with other subtypes of epithelial OVCA including endometrioid (*n* = 3), mucinous (*n* = 3) and clear cell carcinoma (*n* = 3) at early stages (Figure 2D). On the other hand, among non-serous histotypes at early stage, the population of intra-tumoral CISH-expressing cells was lowest (6.423 ± 0.065 in 20,000 µm^2^ area) in endometrioid OVCA (Figure 2D). However, significant differences were not observed in the population of intra-tumoral CISH-expressing cells among different histological subtypes of ovarian carcinoma at late stages (*n* = 5 for HGSC, *n* = 3 for endometrioid, mucinous and clear cell carcinomas) (Figure 2G). Compared to normal, the ratio of signal intensities of CISH expression was significantly high in early stage HGSC (*p* < 0.01) and in late stage HGSC (*p* < 0.0001) (Supplementary Figure S3A).

Immunoblotting showed a very weak, almost undetectable band of approximately 29 kDa for CISH protein in normal ovaries (Figure 2H). Compared to normal, the ratio of signal intensity of CISH expression was significantly high in early stage HGSC (*p* < 0.01) and in late stage HGSC (*p* < 0.0001) (Supplementary Figure S3A). Similarly, the qRT-PCR study showed an approximately two-fold increase in CISH gene expression in early stage HGSC (*p* < 0.05) as compared with normal ovaries and increased five-fold in late stage ovarian HGSC (*p* < 0.05) (Figure 2I).

### 3.3. Development and Progression of OVCA Are Associated with Increased Expression of IL-10

**Figure 3:**
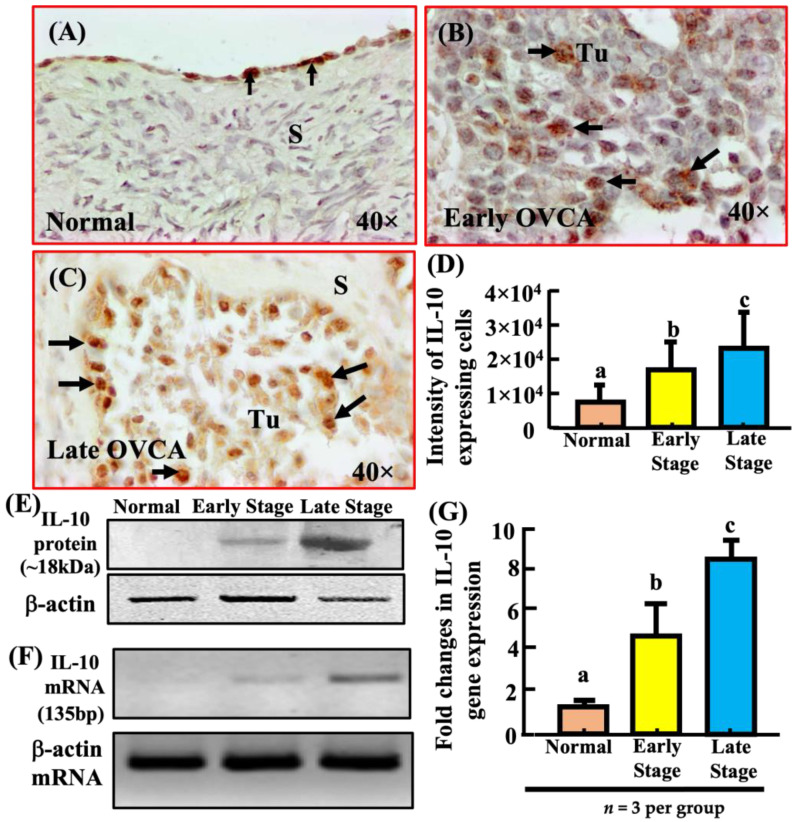
Detection of IL-10-expression in normal ovaries or ovaries with tumor. (**A**) Section of a normal ovary showing IL-10 expression by few ovarian surface epithelial (OSE) cells. (**B**) Section of a high-grade serous carcinoma (HGSC) at early stage showing more IL-10-expressing cells in the tumor. (**C**) Section of a HGSC at late stage showing many IL-10-expressing cells in the tumor. (**D**) Changes in the intensity of IL-10-expression during OVCA development and progression. Compared with normal ovaries (*n* = 7), intensity of IL-10-expression was significantly high in HGSC at early stage (*n* = 5, *p* < 0.05) and increased further in late stage (*n* = 5, *p* < 0.0.001) HGSC. (**E**) Immunoblotting detected a protein band of approximately 18kDa for IL-10. Additional information: Compared to normal, the ratio of signal intensity of IL-10 expression was significantly higher in early stage (*p* < 0.0001) and late stage HGSC (*p* < 0.0001) (Supplementary Figure S3B). (**F**,**G**) As observed for IL-10 protein expression in immunoblotting, semi-quantitative and quantitative RT-PCR showed significant increase in IL-10 mRNA expression in early (*n* = 3, normal vs. early stage, *p* < 0.05) and late stage (*n* = 3, normal vs. late, *p* < 0.0001) ovarian HGSC when compared to normal (*n* = 3). S = Stroma; Tu = Tumor. Values for intensity of IL-10-expression are presented as mean ± SEM in 20,000 µm^2^ area of the tissue and bars (in (**D**,**G**)) with different letters are significantly different. 40× = magnification. Arrows indicate examples of immunopositive IL-10-expressing cells.

In normal ovaries, very few ovarian surface epithelial (OSE) cells showed immunoreactivity for IL-10 expression (Figure 3A). Compared with OSE cells in normal ovaries, malignant cells in ovarian HGSC at early and late stages showed strong immunoreactivity for IL-10 (Figure 3B,C). The immunohistochemical intensity of IL-10 expression in normal ovaries (*n* = 7) was 7.68 × 10^3^ ± 1.1 × 10^3^ in 20,000 µm^2^ area (Figure 3D). In contrast, compared with normal ovaries, the intensity of IL-10 expression was significantly higher in early HGSC (*p* < 0.05, *n* = 5) and increased further in late stage HGSC (*p* < 0.05, *n* = 5, Figure 3D). A similar pattern of IL-10 expression was also detected in other histological subtypes.

Immunoblotting showed a very weak or undetectable band of IL-10 expression in normal ovaries (Figure 3E). On the other hand, signal intensity for IL-10 was moderate for early stage HGSC and stronger for late stage HGSC. Compared to normal, the ratio of signal intensity of IL-10 expression was significantly higher in early stage HGSC (*p* < 0.0001) and late stage HGSC (*p* < 0.0001) (Supplementary Figure S3B). Similarly, semi-quantitative and quantitative RT-PCR showed a significant increase in fold change of IL-10 mRNA expression in early (*n* = 3, normal vs. early, *p* < 0.05) and late stages (*n* = 3, normal vs. late, *p* < 0.0001) of ovarian HGSC when compared to normal (*n* = 3). The IL-15 immunohistochemical staining pattern was similar to IL-10 (Supplementary Figure S4); however, the data are not reported.

### 3.4. Overexpression of CISH and IL-10 during OVCA Development and Progression Are Associated with Increased Expression of GRP78

**Figure 4:**
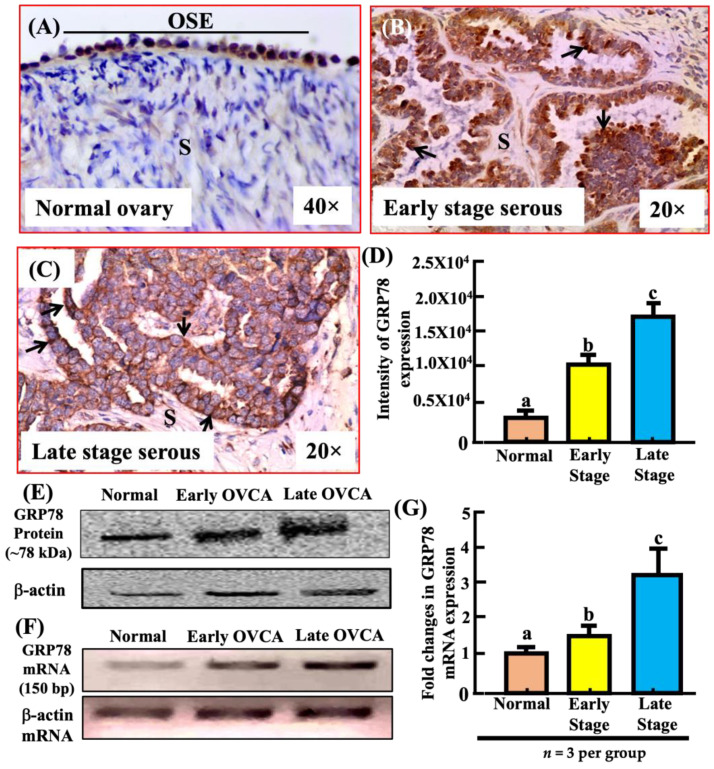
Changes in expression of glucose-regulated protein 78 (GRP78) during the development and progression of ovarian high-grade serous carcinoma (HGSC). (**A**) Section of a normal ovary showing GRP78 expression by ovarian surface epithelial (OSE) cells. (**B**,**C**) Sections of ovarian HGSC at early and late stages showing intense expression of GRP78 on the surface of malignant cells with occasional diffused cytoplasmic staining. (**D**) Changes in the intensity of GRP78 expression during the development and progression of ovarian HGSC. Compared with normal ovaries, the intensity of GRP78 expression was significantly high in HGSC at early stage and increased further in late stage. (**E**) Immunoblotting detected a protein band of approximately 78 kDa for GRP78. Additional information: Compared to normal, the ratio of signal intensity of GRP78 expression was significantly higher in early stage (*p* < 0.0001) and late stage HGSC (*p* < 0.0001) (Supplementary Figure S3C). (**F**,**G**) As compared to normal, semi-quantitative and quantitative RT-PCR showed a significant increase (fold change) in GRP78 mRNA expression in early (normal vs. early, *p* < 0.05, *n* = 3) and late stages (normal vs. late, *p* < 0.05, *n* = 3) of ovarian HGSC. S = Stroma. Values for intensity of GRP78 expression are presented as mean ± SEM in 20,000 µm^2^ area of the tissue and bars (in (**D**,**G**)) with different letters are significantly different. 20×, 40× = magnification. Arrows indicate examples of immunopositive GRP78-expressing cells.

Ovarian surface epithelial cells (OSE) in normal ovaries showed occasional staining for GRP78, a marker of cellular stress (Figure 4A). Compared with normal ovaries, ovarian HGSC at early and late stages showed strong expression for GRP78 (Figure 4B,C). The intensity of GRP78 expression in normal ovaries was 5.82 × 10^3^ ± 0.533 × 10^3^ in 20,000 µm^2^ area (*n* = 7) (Figure 4D). Compared with normal ovaries, the intensity of GRP78 expression was significantly higher in early HGSC (*n* = 5, *p* < 0.05) and increased further in late stage HGSC (*n* = 5, *p* < 0.0001, Figure 4D).

Immunoblotting showed low intensity of expression levels of GRP78 for a band of approximately 78kDa protein in a normal ovary (Figure 4E). Compared to normal, the ratio of signal intensity of GRP78 expression was significantly higher in early stage HGSC (*p* < 0.0001) and late stage HGSC (*p* < 0.0001) (Supplementary Figure S3C). Similar patterns of expression were detected for the GRP78 gene by RT-PCR and qRT-PCR assays (Figure 4F). An approximately 1.5-fold increase in GRP78 gene expression was detected in early stage ovarian HGSC (normal vs. early, *p* < 0.05, *n* = 3) in quantitative RT-PCR as compared with normal ovaries (*n* = 3). The expression increased further in late stage HGSC (normal vs. late, *p* < 0.05, *n* = 3) (Figure 4G).

Correlation studies showed that the population of intra-tumoral CISH-expressing cells was positively correlated with the intensity of IL-10 expression in both early stage ovarian HGSC (r = 0.52, *p* < 0.05, *n* = 5) and late stage ovarian HGSC (r = 0.65, *p* < 0.05, *n* = 5). Similarly, the intensity of immunohistochemical staining of IL-10 was positively correlated with the intensity of immunohistochemical staining of GRP78 in early stage ovarian HGSC (*p* < 0.05, r = 0.43, *n* = 5) and late stage ovarian HGSC (*p* < 0.05, r = 0.52, *n* = 5).

## 4. Discussion

This study reports for the first time that ovarian cancer (OVCA) development and progression is associated with the enhanced expression of CISH, a marker of NK cell exhaustion, and an increased influx of CISH-expressing cells into the tumor. This study further showed that enhancement in the population of CISH-expressing cells was associated with the increased expression of IL-10 (an anti-inflammatory cytokine) and GRP78 (a marker of endoplasmic reticular stress).

This study showed that compared to normal ovaries, the population of CISH-expressing cells was significantly higher in OVCA patients at early and late stages. CISH has been reported as a marker of exhaustion and is mainly expressed by NK cells [36]. NK cells, as members of innate immunity, mount immune responses against developing tumors [37]; however, tumors evade NK cell immunity [38]. It is possible that tumors may induce exhaustion in NK cells and facilitate the evasion of NK cell immunity by the malignant cells. This assumption is supported by the observation that the population of intra-tumoral CISH-expressing cells was significantly higher in OVCA at early and late stages. Thus, the positive association between the population of CISH-expressing cells and tumor progression suggests that induction of CISH expression may be one of the mechanisms of tumor-induced exhaustion of NK cells. However, the mechanisms of tumor-induced CISH expression are not fully understood.

Members of the immune system either kill tumor cells directly or respond to a growing tumor by their secretory products including cytokines [39]. Ovarian tumors, on the other hand, protect themselves from immune attack either by evading immune recognition or suppressing the effector functions of immune cells by tumor-induced soluble factors [40]. Moreover, studies have shown that tumors can transform their microenvironment in their favor by secreting factors including IL-10. IL-10 has been reported both for its anti-tumor and pro-tumor functions [41,42,43,44]. As an anti-inflammatory cytokine, IL-10 prevents tissue damages by regulating and limiting immune responses [45]. This study showed malignant cells in developing tumors expressed anti-inflammatory cytokine IL-10 and its expression increased as the tumor progressed to late stages, suggesting that NK cells in the tumor and its vicinity are persistently exposed to tumor-induced IL-10. In addition, the population of intra-tumoral CISH-expressing cells was also increased in association with the increase in IL-10 expression by malignant cells during the progression of OVCA to late stages. Thus, it is assumed that persistent exposure to tumor-induced IL-10 may exhaust NK cells as reported for IL-15 induced exhaustion [17]. This study also detected increased expression of IL-15 by malignant cells (shown in Supplementary Figure S4 as an example; however, it was not reported in the results). This assumption is also supported by the reports that NK cells express IL-10 receptors on their surface [46]. However, factors involved in increased IL-10 expression by the tumor are not well understood.

This study showed that ovarian tumor development and progression were associated with the increased expression of GRP78 (a marker of cellular stress) by the malignant cells as reported earlier [47]. Cells under stress increase the production of GRP78 to withstand and protect themselves from stressful conditions and death [48]. Malignant cells in tumors are under continuous stress and, as observed in this study, an increase in GRP78 expression has also been reported in several other cancers [47,49,50,51]. Changes in IL-10 expression was positively correlated with CISH and GRP78 during OVCA progression and development. IL-10 has been reported to prevent tissue damages through limiting immune responses [52]. It is possible that ovarian tumors might have maintained persistent expression of IL-10 by increasing the expression of GRP78 and exhausting NK cells by inducing CISH expression and, thus, evading NK cell recognition.

One of the limitations of this study was the smaller sample size; however, the results reported here are based on both immunohistochemical and immunoblotting as well as gene expression studies. In conclusion, this study showed OVCA progression is associated with the increased expression of CISH and intra-tumoral influx of CISH-expressing cells. The increased expression of CISH was associated with the increased expression of anti-inflammatory cytokine IL-10 and a cellular stress marker, GRP78. Overall, the results of this study will be a foundation for a clinical study with larger cohorts and may be useful in developing anti-NK immunotherapies.

## Data Availability

All data supporting the conclusions of this article are included in the article.

## Supplementary Materials

The following supporting information can be downloaded at: https://www.mdpi.com/article/10.3390/biomedicines11020299/s1, Figure S1: Expression of CISH and GRP78 in the normal fimbria of the fallopian tube, Figure S2: Differential staining for the detection of tumor and tumor stroma, Figure S3: Changes in immunoblotting signal intensity in CISH, IL-10 and GRP78 markers, Figure S4: Immunohistochemical detection of IL-15 expression by ovarian high-grade serous carcinoma. Table S1: Antibody Information, Table S2: Sample sizes for CISH, IL-10 and GRP78 markers.
