# Supplementary material for: Expression of CISH, an Inhibitor of NK Cell Function, Increases in Association with Ovarian Cancer Development and Progression
Source: Biomedicines. 2023 Jan 21;11(2):299. doi: 10.3390/biomedicines11020299 (PMC9952877; doi:10.3390/biomedicines11020299)
Supplement: Supplementary file 1 [file biomedicines-11-00299-s001.zip › biomedicines-2157080-supplementary.pdf]

Supplementary Table S1: Antibody Information

Antibody	Catalog Number	Lot Number	Host	Company
**Anti-CISH**	ab191447	GR249409-7	Rabbit	Abcam
**Anti-IL-10**	MABF-2293-100UG	Q3329716	Mouse	Millipore Sigma
**Anti-GRP78**	ab32618	GR3185758-3	Rabbit	Abcam
**Anti-pan Cytokeratin**	ab6401	GR1281-5	Mouse	Abcam
**Anti-CD10**	18008-I-AP	00020124	Rabbit	Proteintech
**Anti-IL-15**	ab109082	GR52126-19	Rabbit	Abcam
**Anti-β Actin**	622102	B347691	Rabbit	BioLegend

Supplementary Table S2:
Sample sizes for CISH, IL-10 and GRP78 markers

Specimen	Marker	Sample Size	Subtype
**Normal**	CISH	n=7	N/A
	IL-10	n=7	N/A
	GRP78	n=7	N/A
**Early stage**	CISH	n=5	HGSC
		n=3	Endometrioid
		n= 3	Mucinous
		n= 3	Clear Cell
	IL-10	n= 5	HGSC
	GRP78	n= 5	HGSC
**Late stage**	CISH	n=5	HGSC
		n=3	Endometrioid
		n=3	Mucinous
		n=3	Clear Cell
	IL-10	n=5	HGSC
	GRP78	n=5	HGSC

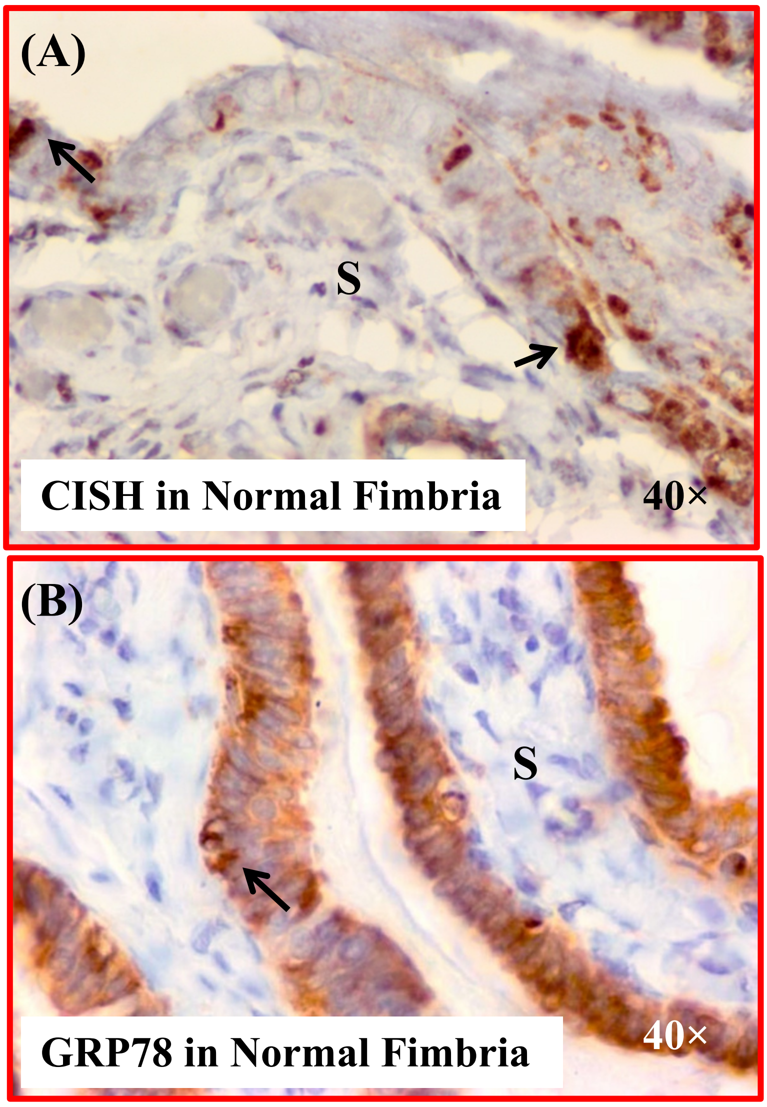

Supplementary Figure S1. Expression of CISH and GRP78 in the normal fimbria of the fallopian tube. **(A)** Section of a normal fimbria from a premenopausal women showing a few CISH-expressing cells. **(B)** Section of a normal fimbria from a premenopausal woman showing GRP78 staining. Fimbrial epithelial cells showed weaker staining for GRP78. S = Stroma, 40× = magnification. Arrows indicate examples of immunostaining.

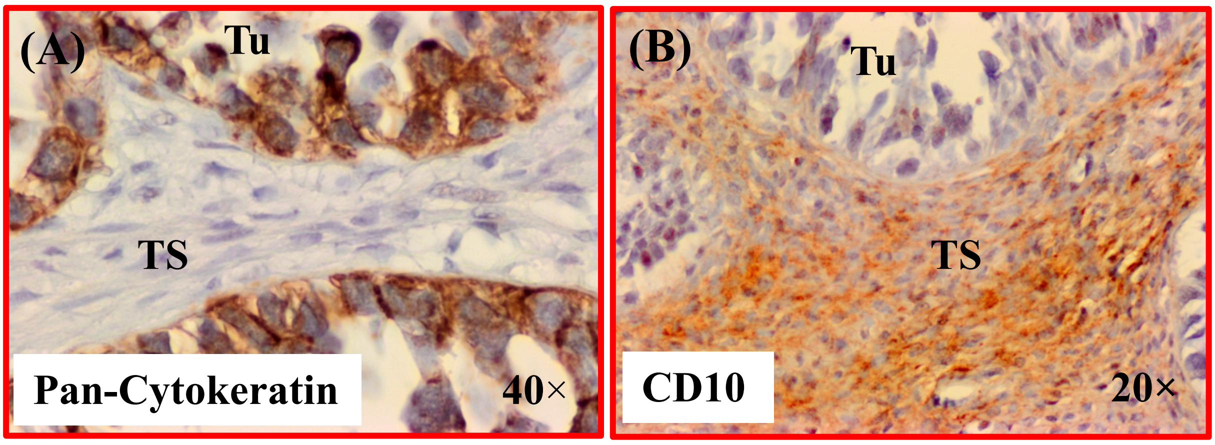

Supplementary Figure S2. Differential staining for the detection of tumor and tumor stroma. **(A)** Malignant cells expressed pan-cytokeratin (an epithelial marker) while stromal cells showed no staining. **(B)** Stromal tissue showed expression for CD10, where malignant cells showed no staining. TS = Tumor stroma, Tu = Tumor. 40× = magnification.

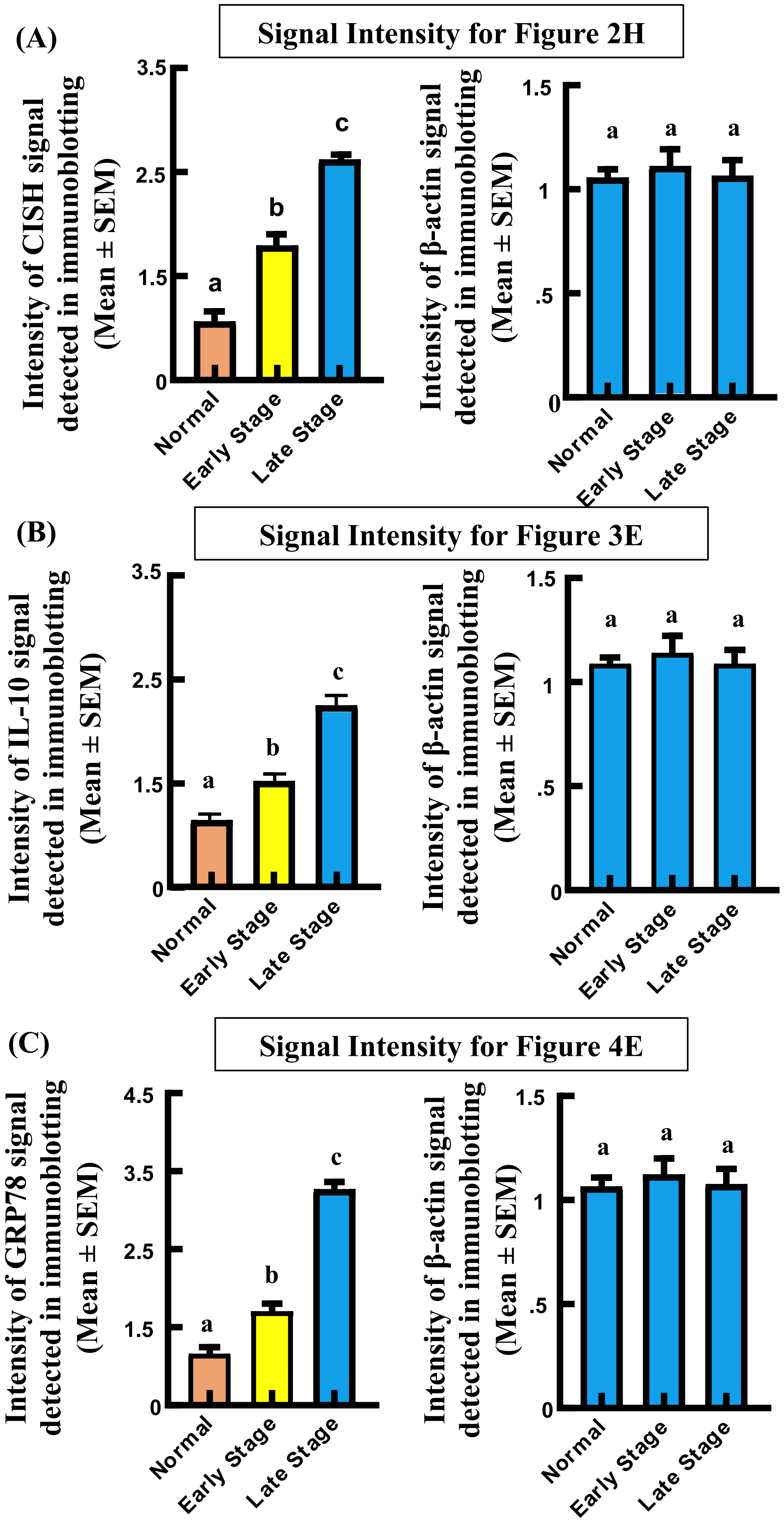

Supplementary Figure S3: Changes in immunoblotting signal intensity in CISH, IL-10 and GRP78 markers. Bar graphs showing ratios of signal intensity of CISH **(A)**, IL-10 **(B)**, GRP78 **(C)**. Ratios of signal intensities were determined as reported previously [33]. Briefly, mean intensity of each group for each marker was divided by their corresponding mean of the normal group (For example, Normal vs. normal (always 1), early stage vs. normal, late stage vs. normal). Signal intensity for all CISH or IL-10 or GRP78 increased significantly, bars with different letters (a, b, c) denote statistical significance.

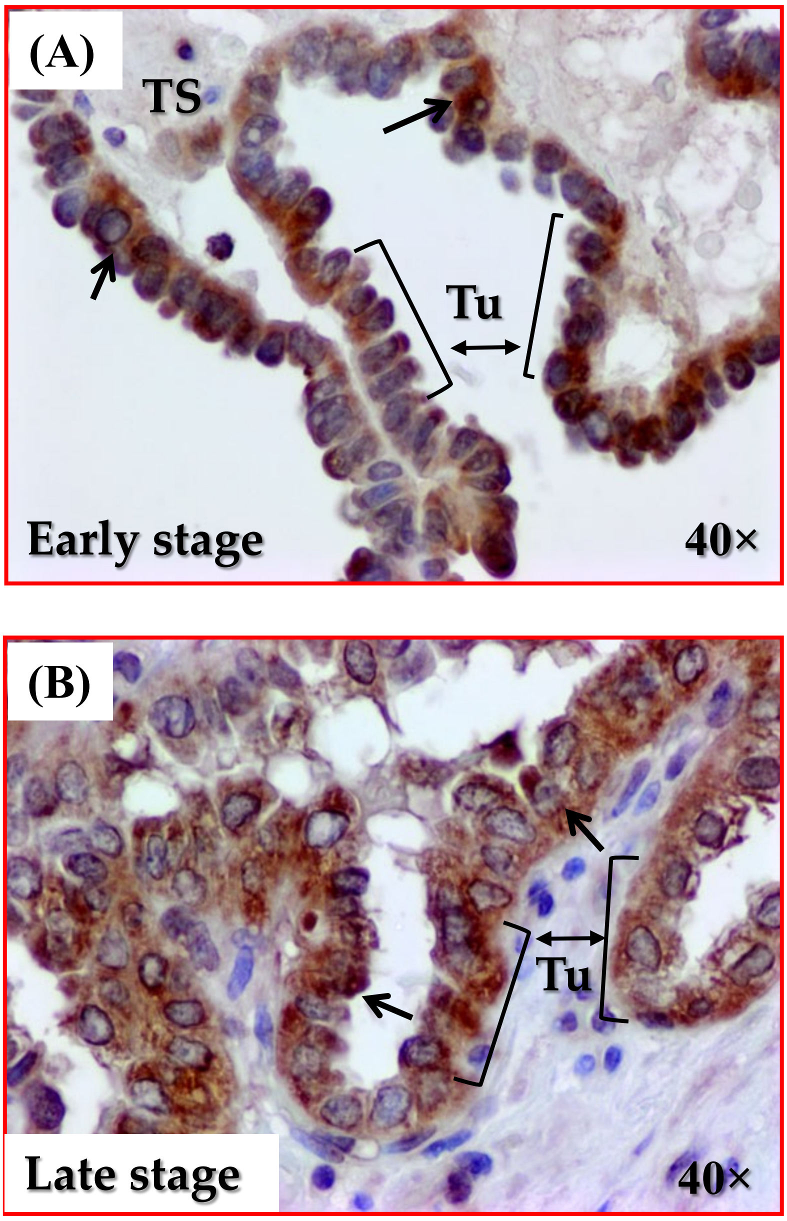

Supplementary Figure S4. Immunohistochemical detection of IL-15 expression by ovarian high-grade serous carcinoma. **(A)** Section of ovarian HGSC at early stage showing IL-15 expression by malignant cells. **(B)** Section of ovarian HGSC showing more cells expressing IL-15. Arrows indicate examples of immunopositive IL-15-expressing cells. Tu= Tumor, TS = Tumor stroma, 40×= magnification.
